# Characterization of Non-Volatile and Volatile in Flat Green Teas Processed by Green, Yellow, and Purple-Colored Leaves Using Multi-Sensory Analysis and Metabolomics

**DOI:** 10.3390/foods15111862

**Published:** 2026-05-24

**Authors:** Yumeng Ding, Yuxin Shen, Lihe Qi, Kai Zhang, Yuxuan Ouyang, Chuan Yue

**Affiliations:** 1 Integrative Science Center of Germplasm Creation in Western China (CHONGQING) Science City, College of Food Science, Southwest University, Chongqing 400715, China; dymying731@163.com (Y.D.); syx050319@163.com (Y.S.); 19172470129@163.com (L.Q.); 18908361167@163.com (Y.O.); 2 Chongqing Agricultural Technology Extension Station, Chongqing 400715, China; xiaocharen@163.com; 3 Chongqing Key Laboratory of Speciality Food Co-Built by Sichuan and Chongqing, Southwest University, Chongqing 400715, China

**Keywords:** teas of special leaf colors, non-volatile compounds, volatiles compounds, flat green tea, GC-E-nose, HS-SPME-GC-MS

## Abstract

Teas processed from specialty-colored tea leaves possess distinctive quality profiles shaped by their volatile and non-volatile compounds, which serve as critical metrics for evaluating tea cultivars. In this study, we comprehensively characterized the quality attributes of flat green teas produced from three tea cultivars—green-leaved ‘FDDB’, yellow-leaved ‘ZH2’, and purple-leaved ‘ZJ’—using an integrated analytical approach including sensory evaluation, widely targeted metabolomics, GC-E-nose, and HS-SPME-GC-MS. Sensory evaluation revealed distinct sensory characteristics among teas processed from the three cultivars with different leaf colors. GC-E-nose analysis further confirmed that the aroma profiles of these tea samples could be clearly distinguished based on leaf color. Metabolomic analysis identified a total of 2050 non-volatile compounds, among which 18 amino acids, 5 phenolic acids, and 4 flavonoids were pinpointed as key contributors to the unique taste profiles of infusions from ZH2 and ZJ teas. Additionally, a total of 1100 volatile compounds were detected, with 94, 75, and 90 key aroma-active compounds identified in FDDB, ZH2, and ZJ teas, respectively. Collectively, in this study, systematic analysis revealed significant differences in both volatile and non-volatile chemical compositions across the three tea cultivars. These findings provide a scientific foundation for understanding the processing suitability and quality formation mechanisms of tea cultivars with distinct leaf colors.

## 1. Introduction

Tea plant [*Camellia sinensis* (L.) O. Kuntze], a perennial plant whose leaves are used to produce various kinds of tea products has significant health benefits for humans. China possesses the world’s richest collection of tea germplasm resources, providing a foundation for breeding distinctive cultivars and developing novel tea products [1,2,3]. In recent years, tea varieties with special leaf colors have attracted increasing attention due to their unique economic potential. Tea leaf colors are typically green, white, yellow, and purple. Leaf color variation affects the metabolite profiles of tea plants, which further influence the sensory quality and flavor of teas. Under identical growing and processing conditions, teas from different leaf color varieties show significant quality differences [4,5,6,7]. For instance, yellow-leaf teas typically contain higher amino acids and lower catechins. This composition contributes to a fresh and umami taste [8,9]. Purple-leaf teas, in contrast, are rich in anthocyanins. They exhibit strong antioxidant activity and a pronounced bitter-astringent profile [10,11].

As a leafy crop, tea quality is largely determined by the metabolic composition of the leaves. Non-volatile compounds such as L-theanine and flavonoids are key contributors to taste [12,13,14], while over 700 volatile metabolites collectively shape the aroma profile of tea infusions [15,16,17,18]. Metabolomics has emerged as a powerful tool for deciphering the chemical basis of tea quality. Widely targeted metabolomics based on UPLC-ESI-MS/MS enables comprehensive profiling of non-volatile compounds, whereas GC-MS/MS and ultra-fast GC-E-nose are commonly applied for volatile aroma analysis [19,20,21,22,23]. Despite extensive research on the chemical and sensory properties of tea cultivars with diverse leaf colors, systematic comparisons of green teas processed from yellow-leaf and purple-leaf varieties remain limited.

In this study, three tea plant cultivars with different leaf colors were used, named ‘Fuding Dabai Tea’ (FDDB, a green leaf variety), ‘Zhonghuang No. 2’ (ZH2, a yellow leaf variety) and ‘Zijuan’ (ZJ, a purple leaf variety), respectively. All were processed into flat green tea using a standardized method. Their non-volatile and volatile profiles were characterized through sensory evaluation, UPLC-ESI-MS/MS, GC-E-nose, and HS-SPME-GC-MS. The findings aim to provide new insights into the characteristic metabolites of specialty leaf-color tea varieties and offer a theoretical basis for the utilization of these unique germplasm resources.

## 2. Materials and Methods

### 2.1. Chemicals

HPLC-grade methanol, acetonitrile, and n-hexane used in the experiments were purchased from Merck (St. Louis, MO, USA). HPLC-grade formic acid was obtained from Aladdin Biochemical & Technology Co., Ltd. (Shanghai, China), and analytical-grade sodium chloride was sourced from Sinopharm Chemical Reagent Co., Ltd. (Shanghai, China). All other chemicals met chromatographic or analytical grade specifications. The 20 mL headspace vials and screw caps fitted with silicone/PTFE septum were purchased from Agilent Technologies Inc. (Palo Alto, CA, USA). Purified water was supplied by Hangzhou Wahaha Group Co., Ltd. (Hangzhou, China).

### 2.2. Tea Samples Processing

The test materials consisted of fresh leaves (one leaf and one bud) from three tea plant varieties. These were harvested in spring 2024 at Gaoyangbifeng Tea Co., Ltd. (Guangyuan City, Sichuan Province). These varieties were named ‘*Camelia sinensis* cv. Fudingdabaicha’ (a green leaf variety), ‘*Camelia sinensis* cv. Zhonghuang No. 2’ (a yellow leaf variety) and ‘*Camelia sinensis* cv. Zijuan’ (a purple leaf variety), and marked as FDDB, ZH2, and ZJ, respectively. All samples were processed into flat-shaped green tea using traditional methods, including withering, fixation, shaping, and drying. The detailed procedures were as follows: Firstly, fresh leaves picked on sunny days were placed in a well-ventilated indoor space for natural withering. This withering process was continued until the leaves exhibited wilting, texture softening, color deepening, a moisture content of 68–72%, and a transition from grassy notes to a fresh fragrance (5–8 h). Then, the withered leaves were pan-fired at 240 °C. As the leaves wilted, the petioles softened, and the color deepened further. Pressure was gradually increased every 30 s. Next, the cut tea leaves were shaped using a flat-bottomed wok. The initial wok temperature was set to 110 °C and maintained for 15 min, during which the humidity was maintained between 20% to produce tight, straight strands. Finally, the wok temperature was adjusted to 90 °C and heating was continued until the moisture content of the leaves dropped to 5–6%.

### 2.3. Sensory Evaluation

Sensory evaluation of tea samples was conducted according to the standard of China (GB/T 23776, 2018 [24]). The intensity values and aroma descriptors of the samples were verified by ten panelists, which were composed of six males and four females with an average age of 30, as previously described by Yue et al. (2023) [25]. Briefly, 200 g of each tea sample was placed on a white square plate to assess the dry tea appearance evaluation. Then, 3 g of each tea sample was brewed with 150 mL of boiling water in a white porcelain cup, and steeped for 4 min. The infusion was filtered into a porcelain bowl and successively evaluated for liquor color, aroma, taste. The intensities of the aroma and taste attributes were scored from 0 to 10, with the intensity ranging from no intensity to high perceptible.

### 2.4. Chromatic Difference Assessment

The color quality of the dry tea leaves was analyzed using a colorimeter apparatus (CM-5, Konica Minolta Investment Co., Ltd., Shanghai, China) equipped with a D65 light source. Color indicators included L*, a*, and b*, representing the luminance (bright: 100, dark: 0), red-green degree (red: +, green: −), yellow-blue degree (yellow: +, blue: −), respectively [26,27]. Each tea sample was measured three times. Ultrapure water was used as the blank control. The final result was the average of the three replicates.

### 2.5. Analysis of Non-Volatile Metabolites

Tea samples were dried using a vacuum freeze dryer (Scientz-100F, Ningbo Scientz Biotechnology Co., Ltd., Ningbo, China). The freeze-dried samples were crushed using a grinder (MM 400, Retsch, Haan, Germany) at a frequency of 30 Hz. Then, 50 mg of sample powder was weighed and mixed with 1.2 mL of pre-cooled (−20 °C) 70% aqueous methanol. The mixture was extracted at 4 °C for 3 h, with vortexing every 30 min. After centrifugation at 12,000 rpm for 3 min, the supernatant was filtered through a microporous membrane (0.22 μm pore size) and subjected to UPLC-ESI-MS/MS analysis, as previously described by Cao et al. (2024) [28]. Quantification was performed using multiple reaction monitoring (MRM) mode. The detailed MRM ion pairs (Q1 and Q3) for representative non-volatile compounds are provided in Table S1. Declustering potential (DP) and collision energy (CE) for individual MRM transitions were optimized. A specific set of MRM transitions was monitored for each period according to the metabolites eluted within that period. Non-volatile metabolites were identified by comparing the mass spectra with the data system library and linear retention index (Metware Biotechnology Co., Ltd., Wuhan, China). For two-group comparisons, differential metabolites were determined by VIP ≥ 1 and |Log_2_FC| ≥ 1.0. VIP values were extracted from OPLS-DA results, which were generated using the MetaboAnalystR package (version 1.0.1). Prior to OPLS-DA, all data were log transform (log_2_) and mean centering (without additional scaling). This preprocessing approach preserves the original variance structure of metabolites, which is appropriate for discriminant analysis when identifying group-specific differential metabolites. To avoid overfitting, a permutation test (200 permutations) was performed. For PCA, unit variance (UV) scaling was applied following the standard protocol of the metabolomics service provider (Metware Biotechnology Co., Ltd., Wuhan, China).

### 2.6. Measurement of Flavor Characteristics Using GC-E-nose

To investigate the volatile fingerprints of tea samples, a GC-E-nose (Alpha M.O.S., Toulouse, France) was used to detect their aroma profiles as previously described by Yang et al. (2022) [29,30]. In brief, 0.5 g of tea sample was placed into a 20 mL sealed glass vial and incubated at 65 °C for 30 min prior to instrumental analysis. High-purity helium was used as the carrier gas at a flow rate of 1 mL/min. Volatile compounds were absorbed by an embedded volatile concentrator (Tenax TA) at 20 °C for 30 s in split mode (10 mL/min), followed by thermal desorption at 240 °C for 30 s. Separation was performed in parallel using a weak polar MXT-5 column and a medium polar MXT-1701 column (20 m × 0.18 mm I.D. × 0.4 μm, Restek Corporation, Bellefonte, PA, USA). The heating program was set as follows: initial temperature was held at 50 °C for 5 s, then ramped to 120 °C at 0.2 °C/s, and finally raised to 250 °C at 0.4 °C/s for 10 s. Both flame ionization detectors were maintained at 260 °C. All analyses were carried out in triplicate.

### 2.7. Volatile Metabolites Detection and rOAV Calculation

Samples were ground into powder in a liquid nitrogen environment. A total of 500 mg of powder was promptly transferred to a 20 mL headspace vial (Agilent, Palo Alto, CA, USA), containing NaCl saturated solution, to inhibit any enzyme reaction. Each sample was extracted three times using fully automated headspace solid-phase microextraction and analyzed by GC–MS as described by Yue et al. (2023) [25]. Qualitative and quantitative analysis of volatiles compounds was performed using an Agilent 8890 GC-7000D MS system (Agilent Technologies Inc., Santa Clara, CA, USA) equipped with a 30 m × 0.25 mm × 0.25 μm DB-5MS (5% phenyl-polymethylsiloxane) capillary column. Helium was used as the carrier gas at a linear flow rate of 1.2 mL/min. The injector port temperature was maintained at 250 °C. The column oven temperature program was as follows: initial temperature of 40 °C held for 3.5 min, followed by a ramp of 10 °C/min to 100 °C, 7 °C/min to 180 °C and 25 °C/min to 280 °C, where it was held for 5 min. Mass spectrometry detection employed 70 eV electron bombard ionization mode. The quadrupole mass detector, ion source, and transmission line temperatures were set to 150 °C, 230 °C, and 280 °C, respectively. Qualitative and quantitative analysis of target analytes were performed using selected ion monitoring (SIM) mode. The quantitative and qualitative ions used for SIM analysis of volatile compounds are provided in Table S2. The contents of volatile metabolites were calculated according to the peak area under the control of an internal standard compound, [2,3,4,5,6-^2^H_5_]-Benzyl Acetate (isoreag, catalog No. IR-20681-250 mg, Canada, CAS: 1398065-57-0, purity: 95%, 98% atom ^2^H). The differential metabolites between two-group comparison were identified based on VIP ≥ 1 and |Log_2_FC| ≥ 1.0. The relative odor activity value (rOAV) was determined as previously described [25]. Compounds with a rOAV > 1 were identified as key aroma-active compounds dominating the overall aroma profile [31,32,33].

### 2.8. Statistical Analysis

To ensure the reliability of the analytical results, both volatile and non-volatile compounds were analyzed with three technical replicates, consisting of three independent extractions and instrumental injections. Metabolomics and volatile compound data processing were carried out using the base package in R (version 4.1.2) and Origin software (2026). Principal component analysis (PCA), cluster analysis, and orthogonal partial least squares discrimination analysis (OPLS-DA) were conducted as previously described [25]. Hierarchical cluster analysis (HCA) was performed using the Metware Cloud platform (https://cloud.metware.cn).

## 3. Results and Discussion

### 3.1. Sensory Evaluation of Three Teas

**Figure 1:**
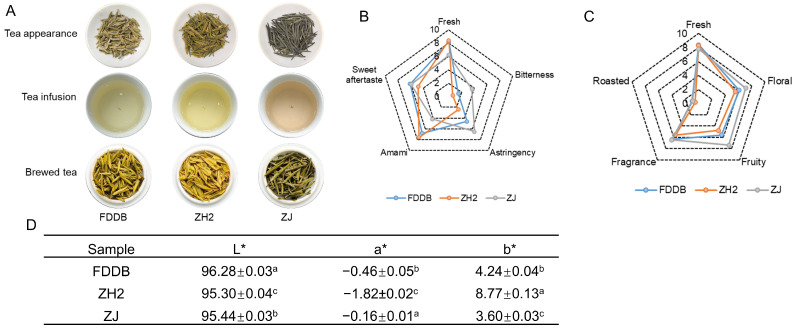
Sensory evaluation of three tea samples processed from FDDB, ZH2, and ZJ, respectively. (**A**) Tea appearance, tea infusion and brewed teas of tea samples; (**B**) Tea taste scores; (**C**) Tea aroma scores; (**D**) Tea infusion colors evaluation, different superscript letters (a, b, c) in the same column indicate significant differences among samples (*p* < 0.05).

In this study, the sensory quality of teas processed from green (FDDB), yellow (ZH2), and purple (ZJ) colors tea leaves were evaluated. As shown in Figure 1A, the tea appearance, tea infusion and brewed teas exhibited high quality, and were distinct from each other, indicating that these three cultivars are suitable for green tea processing. Moreover, ZH2 had the highest umami and fresh taste and lowest bitterness, and FDDB had the highest sweet aftertaste, whereas ZJ exhibited the highest astringency and bitterness and lowest umami taste (Figure 1B). Among these teas, ZJ had the highest floral and fruity aroma (Figure 1C). Color is an important indicator of tea product quality and acceptability. The infusion color of the three samples varied significantly (Figure 1D). The color quality was evaluated based on three indicators: lightness (L*), greenness (−a*), and yellowness (b*). The L* values of FDDB were obviously higher than ZH2 and ZJ, demonstrating better lightness. The |−a| values of FDDB and ZH2 were significantly higher than that of ZJ. And ZH2 exhibited the highest b value. These results suggest that leaf color significantly influences the infusion color characteristics of tea.

### 3.2. Non-Volatile Metabolite Profiling of Three Teas Processed from Different Leaf Colors

#### 3.2.1. Identification of Non-Volatile Compounds

**Figure 2:**
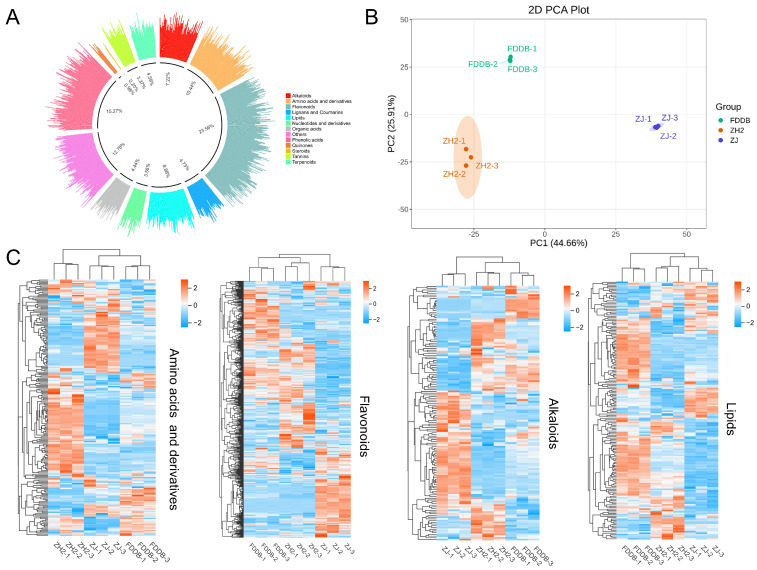
Overview of non-volatile metabolites. (**A**) Types and content of non-volatile metabolites; (**B**) PCA score plot; (**C**) Heatmap of amino acids and derivatives, flavonoids, alkaloids, and lipids in three tea samples.

A total of 2050 non-volatile metabolites were identified via UPLC-MS/MS-based widely targeted metabolomics. These were primarily classified as flavonoids (23.56%), phenolic acids (15.27%), amino acids and their derivatives (10.44%), and lipids (8.88%) (Figure 2A). The MS/MS spectra and UPLC-MS/MS chromatograms of 30 representative non-volatile compounds (level 1) are shown in Figures S1 and S2. PCA revealed clear separation among the three tea samples (Figure 2B), with PC1 and PC2 explaining 37.35% and 25.88% of the variance, respectively, indicating substantial metabolic divergence [34]. Hierarchical clustering heatmaps further highlighted distinctive accumulation patterns of amino acids, flavonoids, alkaloids, and lipids among FDDB, ZH2, and ZJ (Figure 2C).

#### 3.2.2. Analysis of Critical Differential Non-Volatile Metabolites

**Figure 3:**
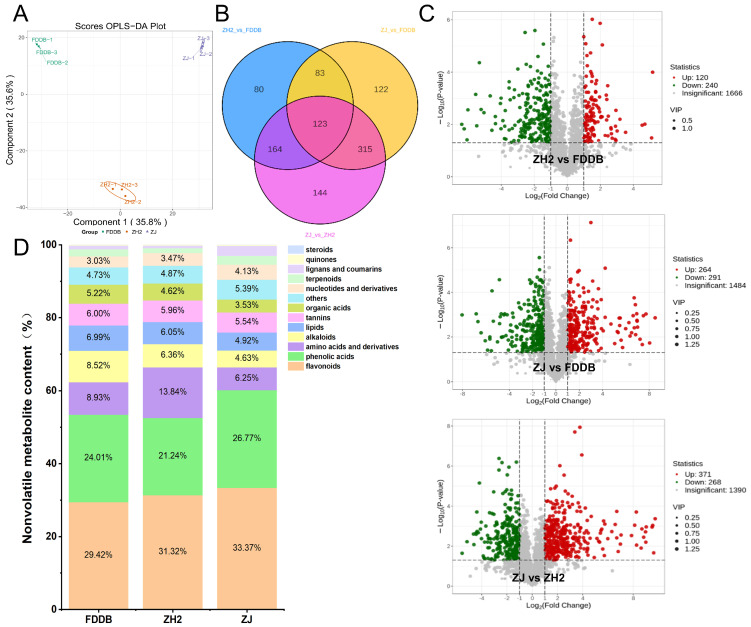
Differentially expressed non-volatile metabolites analysis. (**A**) OPLS-DA score plot; (**B**) Venn plot showing pairwise comparisons of differential non-volatile metabolites; (**C**) Volcano plot showing the number of metabolites differing between ZH2 vs. FDDB, ZJ vs. FDDB, and ZJ vs. ZH2; (**D**) Proportional composition of differential non-volatile compounds across three tea samples.

To evaluate the differential non-volatile compounds among three tea samples, the OPLS-DA method was utilized to examine the distinctions among samples (Figure 3A). The results exhibited distinct variations in the tea samples, which was similar to the findings obtained from PCA. After conducting 200 permutation tests, the values of Q^2^ exceeded 0.9 at *p* < 0.05, demonstrating a high level of reliability without overfitting (Figure S3). To further explore metabolic differences among FDDB, ZH2, and ZJ, the differential metabolites among the three tea samples were identified by calculating the VIP values and log_2_FC, with screening criteria set as VIP ≥ 1 and |log_2_FC| ≥ 1 [25,35]. Based on these standards, 123 non-volatile compounds were overlapped among three tea samples (Figure 3B), potentially representing major contributors to their flavor. Moreover, pairwise comparisons revealed 360 differential metabolites in ZH2 vs. FDDB (including 120 up-regulated and 240 down-regulated), 555 in ZJ vs. FDDB (including 264 up-regulated and 291 down-regulated), and 639 in ZJ vs. ZH2 (including 371 up-regulated and 268 down-regulated) (Figure 3C). The enhanced umami and sweet taste of ZH2 infusion may be attributed to the accumulation of 18 distinctive amino acids, including L-arginine, L-glutamic acid, and cycloleucine. By contrast, the pronounced bitterness and astringency of ZJ infusion likely results from its higher contents of (1′R,3R,5R,8′S)-dihydrophaseic acid-O-β-D-glucoside and other five phenolic acids as well as cyanidin 3-xyloside and other four flavonoids compared.

As shown in Figure 3D, FDDB was characterized by higher levels of organic acids and alkaloids, contributing to its rich mouthfeel. ZH2 accumulated significantly higher amino acids and their derivatives, consistent with the fresh and mellow taste of its infusion, as previously reported for yellow-leaf teas [36,37,38]. By contrast, ZJ showed the highest levels of flavonoids, phenolic acids, and nucleotides, with flavonoids known to impart bitterness and astringency, a feature commonly observed in purple-leaf teas [39,40,41,42]. These findings indicate that the distinct sensory characteristics of flat green teas from FDDB, ZH2, and ZJ are primarily shaped by cultivar-specific differences in the composition and abundance of non-volatile metabolites.

### 3.3. Volatile Metabolite Profiling of Three Teas Processed from Different Leaf Colors

#### 3.3.1. GC-E-nose Analysis

**Figure 4:**
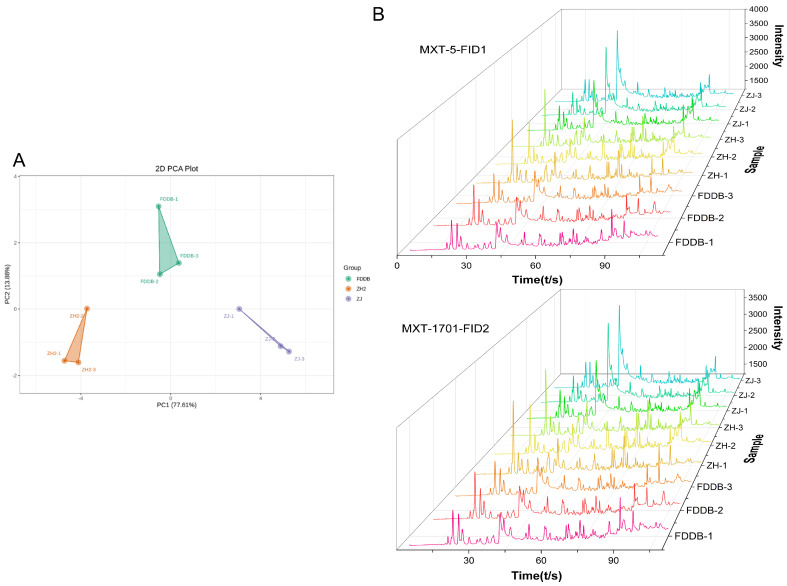
GC-E-nose analysis. (**A**) PCA score plot; (**B**) Fingerprint spectra of three tea samples on MXT-5-FID1 and MXT-1701 columns.

To further investigate the difference in aroma profiles among tea samples, GC-E-nose was used to detect their volatile compounds. PCA of GC-E-nose data showed distinct clustering of FDDB, ZH2, and ZJ, with PC1 and PC2 accounting for 77.61% and 13.88% of the total variance, respectively (Figure 4A). The tight clustering of biological replicates confirmed high reproducibility. Fingerprint spectra and radar plots revealed significant differences in volatile profiles across the three samples on both MXT-5 and MXT-1701 columns (Figure 4B), indicating that leaf color critically influences volatile composition.

#### 3.3.2. Qualitative and Quantitative Analysis of Volatile Compounds

**Figure 5:**
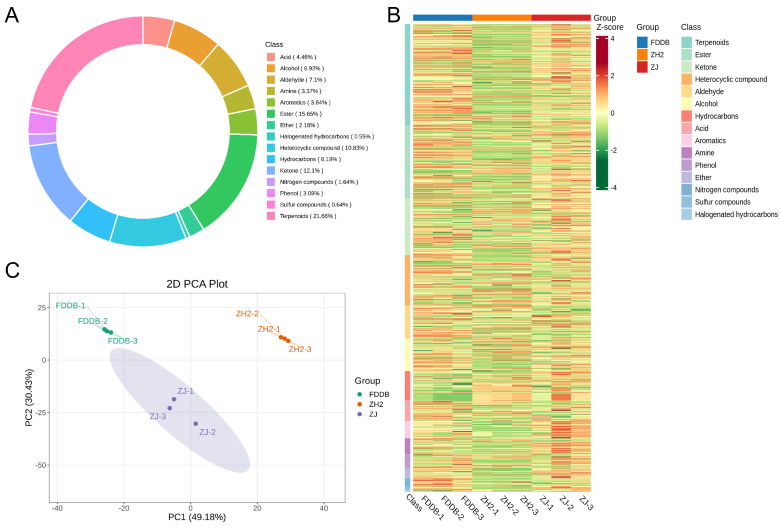
Overview of volatile metabolites. (**A**) Types and content of volatile metabolites; (**B**) Heatmap of volatile compounds; (**C**) PCA score plot.

Aroma, influenced by multiple volatile compounds, serves as a crucial criterion for evaluating tea quality [43,44]. In this study, HS-SPME-GC-MS was used to analyze the aroma profiles of three tea samples, identifying over 1100 volatile compounds. Among these compounds, terpenes exhibited the highest content, accounting for 21.66% of total volatiles, followed by esters, ketones, heterocyclic compounds, and aldehydes, representing 15.65%, 12.10%, 10.83%, and 7.10%, respectively (Figure 5A,B), and this results aligned with the characteristic profile of volatile compounds present in green teas [22,45,46]. The MS/MS spectra and GC-MS/MS chromatograms of 30 key volatile compounds are provided in Figures S4 and S5. Based on these components, we conducted PCA model analysis and showed that the first and second principal components explained 59.70% and 20.31% of the variance (Figure 5C). A clear separation trend was observed among the three tea samples in terms of volatile substance composition, indicating significant differences in the accumulation of volatile metabolites among the three groups.

#### 3.3.3. Identification of Differential Volatile Compounds

**Figure 6:**
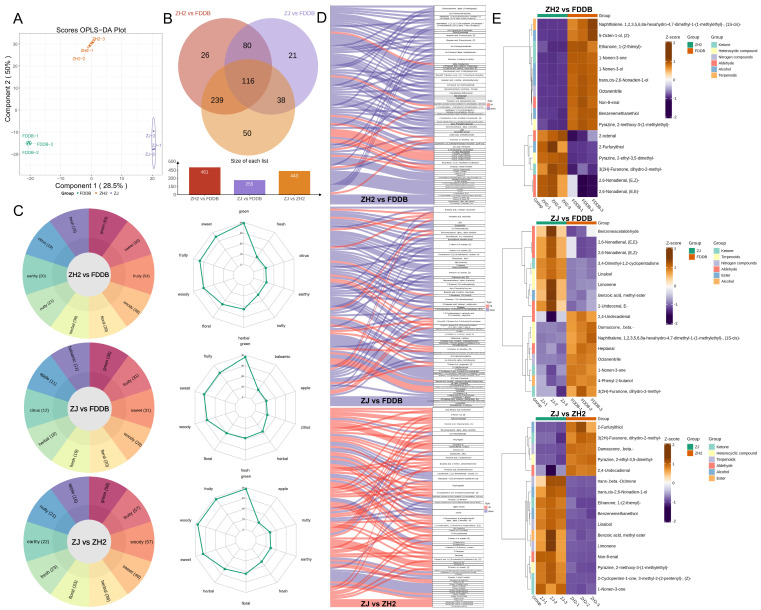
Volatile metabolites analysis. (**A**) OPLS-DA score plot; (**B**) Venn diagram showing pairwise comparisons of differential volatile metabolites; (**C**) Floral profile wheel and radar diagram; (**D**) Sanky diagram. (**E**) Heatmaps showing the amount of differential volatile compounds in different combinations: ZH2 vs. FDDB, ZJ vs. FDDB, and ZJ vs. ZH2.

OPLS-DA based on the 1100 volatile compounds revealed clear separation according to leaf color (Figure 6A). Ultimately, 116 differentially volatile components (VIP > 1) were identified in the study samples, such as (+)-Dihydrocaryone, cis-Dihydrocaryone, o-Decylhydroxylamine, 4-Hexenoic acid acetate, 10-undecylenol, (E)-3-hexenoic acid, trans-2-hexenyl acetateand (E)-3-hexenyl acetate, etc. These compounds have been identified as differential compounds in numerous studies and primarily contribute to characteristics such as the fresh aroma of tea [47,48,49]. Pairwise comparisons identified 461 differential metabolites between ZH2 and FDDB, 443 between ZH2 and ZJ, and only 255 between ZJ and FDDB (Figure 6B). As shown in Figure 6C, ZH2 and ZJ exhibited more pronounced green, fruity, and sweet aromas compared to FDDB, with ZJ showing additional woody aroma. Furthermore, comparative analysis using Sankey diagrams of volatile metabolites confirmed that the flat green teas processed from FDDB, ZH2, and ZJ possessed clearly distinct aroma profiles (Figure 6D). Figure 6E illustrates the expression characteristics of the differential volatile compounds in different comparison groups.

#### 3.3.4. Key Aroma-Active Volatiles in Three Tea Samples with Different Color

It is noteworthy that while over 700 volatile compounds have been identified in tea, only a small number of volatile metabolites in leaves are crucial to the overall aroma characteristic [18,46]. rOAV is used to quantify and evaluate the contribution of various volatile compounds to the overall aroma profile of a sample. According to established criteria in tea flavor research, volatile compounds with an rOAV ≥ 1 are defined as key aroma-active components and those with higher rOAVs exert a more prominent influence on the overall aromatic profile of tea [25,50,51,52]. Based on these criteria, we identified 94, 75, and 90 key aroma-active volatile compounds in FDDB, ZH2, and ZJ, respectively (Table 1). Among these, 1-nonen-3-one, 3(2H)-Furanone, dihydro-2-methyl-, Pyrazine, 2-methoxy-3-(1-methylethyl)- and 54 other compounds were common to all three cultivars. These aroma compounds likely constitute the key components shaping the overall aroma profile of the three flat green tea samples. Interestingly, 3-Buten-2-one, 4-(2,6,6-trimethyl-1-cyclohexen-1-yl)-(floral), Heptanal (fresh) and beta-Damascone (fruity) had been regarded as key specific aroma compounds in FDDB, enhancing its floral, fresh and fruity aromas. Additionally, ZH2 accumulated lower abundances of 4-Phenyl-2-butanol (floral), 5-Octen-1-ol, (Z)- (floral), 3(2H)-Furanone (green), dihydro-2-methyl- (sweet) and Pyrazine, 2-ethyl-3,5-dimethyl- (green), while ZJ showed higher enrichment in 2,6-Nonadienal, (E,Z)- (floral and woody), 2,6-Nonadienal, (E,E)- (floral) and 2-Nonenal (E)- (fruity). This compositional difference aligns with the sensory evaluation results, demonstrating that ZJ displayed the most pronounced floral and fruity notes compared with FDDB and ZH2. These findings had shown that tea cultivars with different leaf colors play a critical role in shaping the volatile metabolome and the sensory quality of tea.

## 4. Conclusions

This study comprehensively characterized the distinctive quality attributes of flat green teas produced from FDDB, ZH2, and ZJ cultivars through an integrated analytical approach encompassing sensory evaluation, GC-E-nose, widely targeted metabolomics, HS-SPME-GC-MS, and rOAV analysis. A total of 2050 non-volatile metabolites were identified across the three tea samples, with flavonoids (483), phenolic acids (313), and amino acids and their derivatives (214) representing the most abundant classes. Comparative analysis of differential metabolites revealed 902, 895, and 1180 differential metabolites in the pairwise comparisons of ZH2 vs. FDDB, ZJ vs. FDDB, and ZH2 vs. ZJ, respectively. These differential metabolites were primarily categorized as flavonoids, phenolic acids, and amino acids and their derivatives. GC-E-nose was verified as an effective tool for the rapid discrimination of tea samples, providing a valuable complement to sensory evaluation. Furthermore, a total of 1100 volatile compounds were identified in the three teas, including 238 terpenoids, 173 esters, 133 ketones, and 119 heterocyclic compounds. Among these, 94, 75, and 90 key aroma-active volatiles (rOAV ≥ 1) were detected in FDDB, ZH2, and ZJ, respectively. Interestingly, FDDB was characterized by 3-Buten-2-one, 4-(2, 6, 6-trimethyl-1-cyclohexen-1-yl)-, Heptanal and beta-Damascone; ZH2 showed lower abundances of 4-Phenyl-2-butanol and 3(2H)-Furanone, and ZJ was enriched in 2,6-Nonadienal and 2-Nonenal. This study provides profound insights into the unique quality foundations of teas processed from cultivars with special leaf colors, laying a scientific foundation for the further development of specialty-colored tea cultivars and the optimization of tea processing techniques to enhance tea quality.

## Figures and Tables

**Table 1:** The volatile compounds with rOAV ≥ 1 in three tea samples.

ID	Name	Threshold µg/g	CAS	Odor Description	rOAV		
					FDDB	ZH2	ZJ
1	1-Nonen-3-one	0.000001	24415-26-7	pungent, mushroom	40,096.99	9761.00	27,229.38
2	3(2H)-Furanone, dihydro-2-methyl-	0.000005	3188-00-9	sweet, solvent, bread, buttery, nutty	38,295.24	58,759.88	25,651.94
3	Pyrazine, 2-methoxy-3-(1-methylethyl)-	0.000002	25773-40-4	beany, pea, earthy, chocolate, nutty	11,019.18	2004.43	11,172.10
4	3-Buten-2-one, 4-(2,6,6-trimethyl-1-cyclohexen-1-yl)-	0.000007	14901-07-6	floral, woody, sweet, fruity, berry, tropical, beeswax	5238.38	3863.68	5283.60
5	Beta-Damascone	0.000002	23726-91-2	fruity, floral, berry, plum, black currant, honey, rose, tobacco	1715.75	2874.49	345.13
6	1-Hexen-3-one	0.00002	1629-60-3	cooked, vegetable, metallic	1012.99	1726.14	1302.12
7	Octanenitrile	0.00013	124-12-9	fatty, aldehydic, green	794.53	152.81	146.98
8	Non-8-enal	0.0002	39770-04-2	smoky, plastic	724.57	82.59	715.63
9	Benzenemethanethiol	0.0000035	100-53-8	sharp, alliaceous, onion, sulfury, garlic, horseradish, minty, coffee	722.21	129.97	561.11
10	4-Heptenal, (Z)-	0.000025	6728-31-0	oily, fatty, green, dairy, milky, creamy	444.89	409.72	280.19
11	2,6-Nonadienal, (E,Z)-	0.00001	557-48-2	cucumber, green	193.93	724.73	773.17
12	2-Cyclopenten-1-one, 3-methyl-2-(2-pentenyl)-, (Z)-	0.00026	488-10-8	woody, herbal, floral, spicy, jasmin, celery	326.63	71.37	377.12
13	2,4-Undecadienal	0.00001	13162-46-4	green, buttery, spicy, baked, fruity, fatty, aldehydic, chicken	191.81	289.56	78.22
14	trans-.beta.-Ionone	0.0002	79-77-6	dry, powdery, floral, woody, orris	183.34	135.23	184.93
15	3-Octen-2-one	0.00003	1669-44-9	earthy, spicy, herbal, sweet, mushroom, hay, blueberry	175.27	99.05	190.42
16	Ethanone, 1-(2-thienyl)-	0.001	88-15-3	sulfury, nutty, hazelnut, walnut	95.81	4.99	86.39
17	Cyclohexanone, 2,2,6-trimethyl-	0.0001	2408-37-9	pungent, thujone, labdanum, honey, cistus	60.79	51.60	61.55
18	2-octenal	0.0002	2363-89-5	fatty, green, herbal	57.82	82.16	65.23
19	Dodecanenitrile	0.00009	2437-25-4	citrus, orange, peel, metallic, spicy	49.64	53.91	61.66
20	3,5-Octadien-2-one, (E,E)-	0.0005	30086-02-3	fruity, green, grassy	28.96	14.86	22.28
21	2-Nonenal, (E)-	0.00008	18829-56-6	fatty, green, cucumber, aldehydic, citrus	29.11	42.26	49.13
22	3-mercapto-2-pentanone	0.0007	67633-97-0	sulfury, metallic, roasted, onion, horseradish, potato	26.30	23.27	16.61
23	6-Nonenal, (Z)-	0.00014	2277-19-2	green, cucumber, melon, cantaloupe, honeydew, waxy, vegetable, orris, violet, leafy	24.51	36.66	34.77
24	2-Nonenal	0.0001	2463-53-8	fatty, green, waxy, cucumber, melon	23.29	33.80	39.30
25	trans,cis-2,6-Nonadien-1-ol	0.001	28069-72-9	green, cucumber, oily, violet, leafy	21.70	4.91	18.14
26	Pyrazine, 2-ethyl-3,5-dimethyl-	0.00004	13925-07-0	burnt, almond, roasted, nutty, coffee	23.10	157.52	38.00
27	1-Nonen-3-ol	0.001	21964-44-3	oily, creamy, green, earthy, mushroom	19.06	1.82	16.55
28	Naphthalene, 1,2,3,5,6,8a-hexahydro-4,7-dimethyl-1-(1-methylethyl)-, (1S-cis)-	0.0015	483-76-1	thyme, herbal, woody, dry	17.92	1.24	2.70
29	2-Furfurylthiol	0.000006	98-02-2	sulfury, roasted, coffee, oily, fatty, burnt, smoky	13.68	84.67	14.27
30	Heptanal	0.0028	111-71-7	fresh, aldehydic, fatty, green, herbal, wine, ozonous	13.17	12.10	7.95
31	2,4-Decadienal, (E,E)-	0.00007	25152-84-5	dusty, waxy, oily, soapy	11.97	13.37	11.58
32	2-Undecenal, E-	0.00078	53448-07-0	fresh, fruity, citrus, orange, peel	9.49	15.48	14.52
33	.beta.-Myrcene	0.015	123-35-3	musty, balsamic, spice	9.16	5.38	8.43
34	5-Octen-1-ol, (Z)-	0.002	64275-73-6	green, melon, watery, watermelon, earthy, mushroom, violet, leafy, fishy, soapy	9.03	2.49	7.36
35	2,6-Nonadienal, (E,E)-	0.0005	17587-33-6	fresh, citrus, green, cucumber, melon	3.88	14.49	15.46
36	Benzoic acid, methyl ester	0.00052	93-58-3	phenol, wintergreen, almond, floral, canga	7.16	3.44	14.91
37	Linalool	0.006	78-70-6	floral, green	6.81	3.30	14.66
38	4-Phenyl-2-butanol	0.0043	2344-70-9	floral, peony, foliage, sweet, mimosa, heliotrope	6.28	2.04	2.30
39	Nonanal	0.001	124-19-6	aldehyde, citrus, orange peel	5.84	5.09	9.76
40	(2S,4R)-4-Methyl-2-(2-methylprop-1-en-1-yl)tetrahydro-2H-pyran	0.0002	3033-23-6	rose, cortex, green, floral, geranium, powdery, metallic	5.92	8.90	7.78
41	2H-Pyran, tetrahydro-4-methyl-2-(2-methyl-1-propenyl)-	0.0002	16409-43-1	sweet, floral, aromatic, rose, fresh, bay, leafy	5.92	8.90	7.78
42	3,4-Dimethyl-1,2-cyclopentadione	0.017	13494-06-9	sweet, maple, caramel, sugar, fenugreek, licorice	4.83	6.89	10.61
43	Benzeneacetaldehyde	0.0063	122-78-1	floral, honey, rose, cherry	4.51	2.29	7.50
44	2-Octenal, (E)-	0.003	2548-87-0	fresh, cucumber, fatty, green, herbal, banana, waxy, leafy	3.85	5.48	4.35
45	Limonene	0.01	138-86-3	citrus, herbal, terpene, camphor	3.63	1.35	9.20
46	Oxazole, trimethyl-	0.005	20662-84-4	nutty, nut skin, roasted, wasabi, shellfish, mustard, vegetable	3.51	4.32	4.34
47	3-Nonanone	0.017	925-78-0	caramel, spicy, sweet	3.21	2.09	3.43
48	trans-.beta.-Ocimene	0.034	3779-61-1	sweet, herbal	3.20	1.65	4.23
49	Bicyclo[2.2.1]heptan-2-ol, 1,7,7-trimethyl-, (1S-endo)-	0.048	464-45-9	pine, woody, camphor	1.92	2.53	2.41
50	Naphthalene, 2-methyl-	0.004	91-57-6	sweet, floral, woody	2.52	2.90	3.50
51	Butanoic acid, butyl ester	0.028	109-21-7	fruity, banana, pineapple, green, cherry, tropical fruit, ripe fruit, juicy fruity	2.83	5.05	3.48
52	trans-Rose oxide	0.0005	876-18-6	floral	2.37	3.56	3.11
53	2-n-Butyl furan	0.005	4466-24-4	mild, fruity, wine, sweet, spicy	2.10	1.95	1.25
54	1,3-Dithiolo[4,5-b]furan, tetrahydro-3a-methyl-	0.006	67411-25-0	boiled, milky, chicken, cooked beef, rubbery, sulfury, thiamin	1.90	2.83	2.34
55	2-Cyclopenten-1-one, 3-ethyl-2-hydroxy-	0.052	21835-01-8	strong, caramel	1.58	2.25	3.47
56	1,3-Cyclohexadiene-1-carboxaldehyde, 2,6,6-trimethyl-	0.003	116-26-7	fresh, herbal, phenol, metallic, rosemary, tobacco, spicy	1.68	2.41	1.32
57	Naphthalene, 1-methyl-	0.008	90-12-0	naphthyl, chemical, medicinal, camphor	1.26	1.45	1.75

## Data Availability

The original contributions presented in this study are included in the article/Supplementary Materials. Further inquiries can be directed to the corresponding author.

## Supplementary Materials

The following supporting information can be downloaded at: https://www.mdpi.com/article/10.3390/foods15111862/s1, Figure S1: The MS/MS spectra of 30 representative non-volatile compounds (level 1). Figure S2: The UPLC-MS/MS chromatogram of 30 representative non-volatile compounds (level 1). Figure S3: Permutation test of the OPLS-DA model of the non-volatile compounds; (A) ZH2 vs. FDDB; (B) ZJ vs. FDDB; (C) ZJ vs. ZH2. Figure S4: The MS/MS spectra of 30 representative volatile compounds. Figure S5: The GC-MS/MS chromatogram of 30 representative volatile compounds. Table S1: The MRM ion pairs (Q1 and Q3) for non-volatile compounds. Table S2: The SIM ion pairs (Quantitative and Qualitative) for volatile compounds.
