# Supplementary material for: Characterization of Non-Volatile and Volatile in Flat Green Teas Processed by Green, Yellow, and Purple-Colored Leaves Using Multi-Sensory Analysis and Metabolomics
Source: Foods. 2026 May 24;15(11):1862. doi: 10.3390/foods15111862 (PMC13256947; doi:10.3390/foods15111862)
Supplement: Supplementary file 1 [file foods-15-01862-s001.zip › foods-4299727-supplementary.pdf]

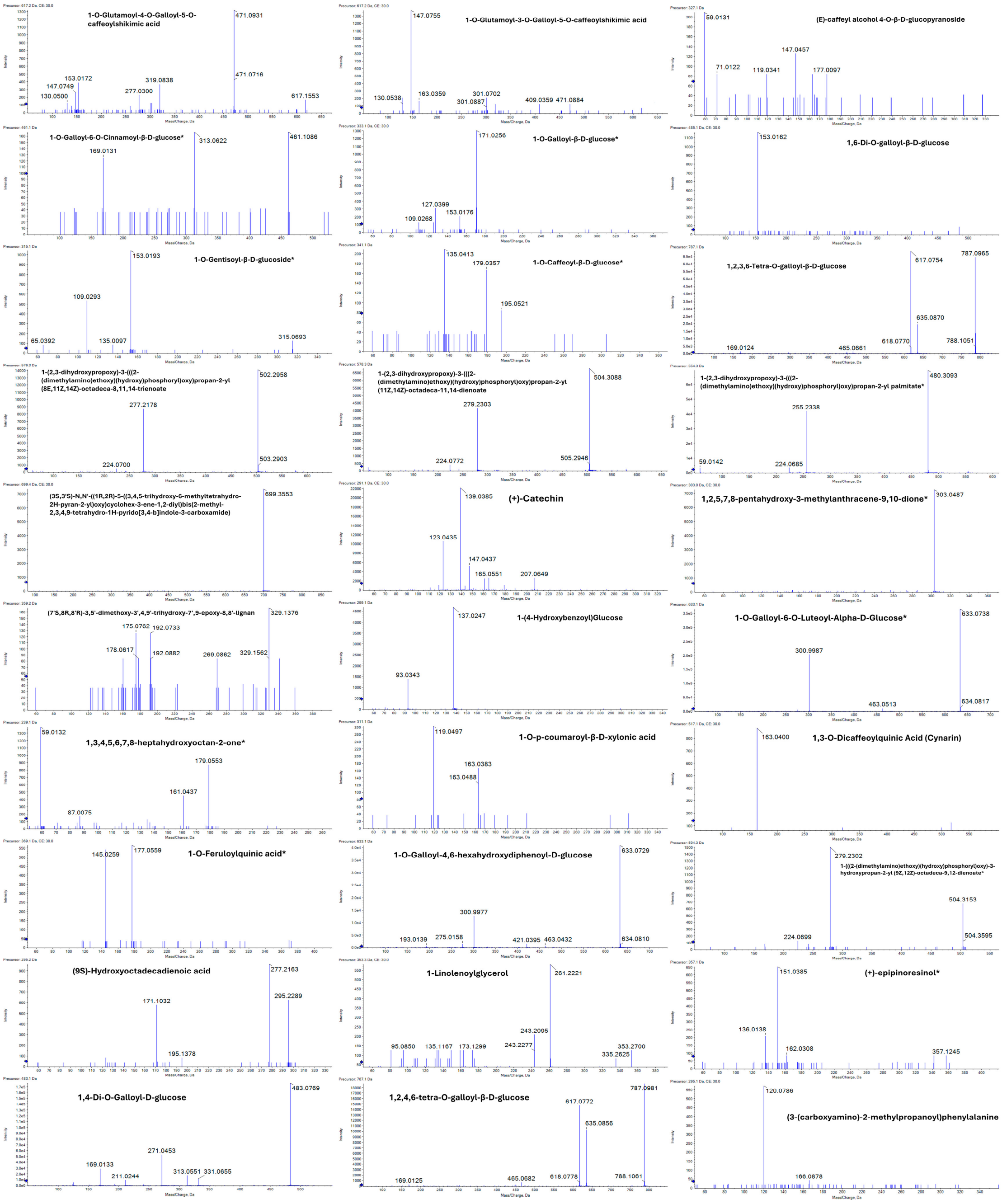

Figure S1: The MS/MS spectra of 30 representative non-volatile compounds (level 1).

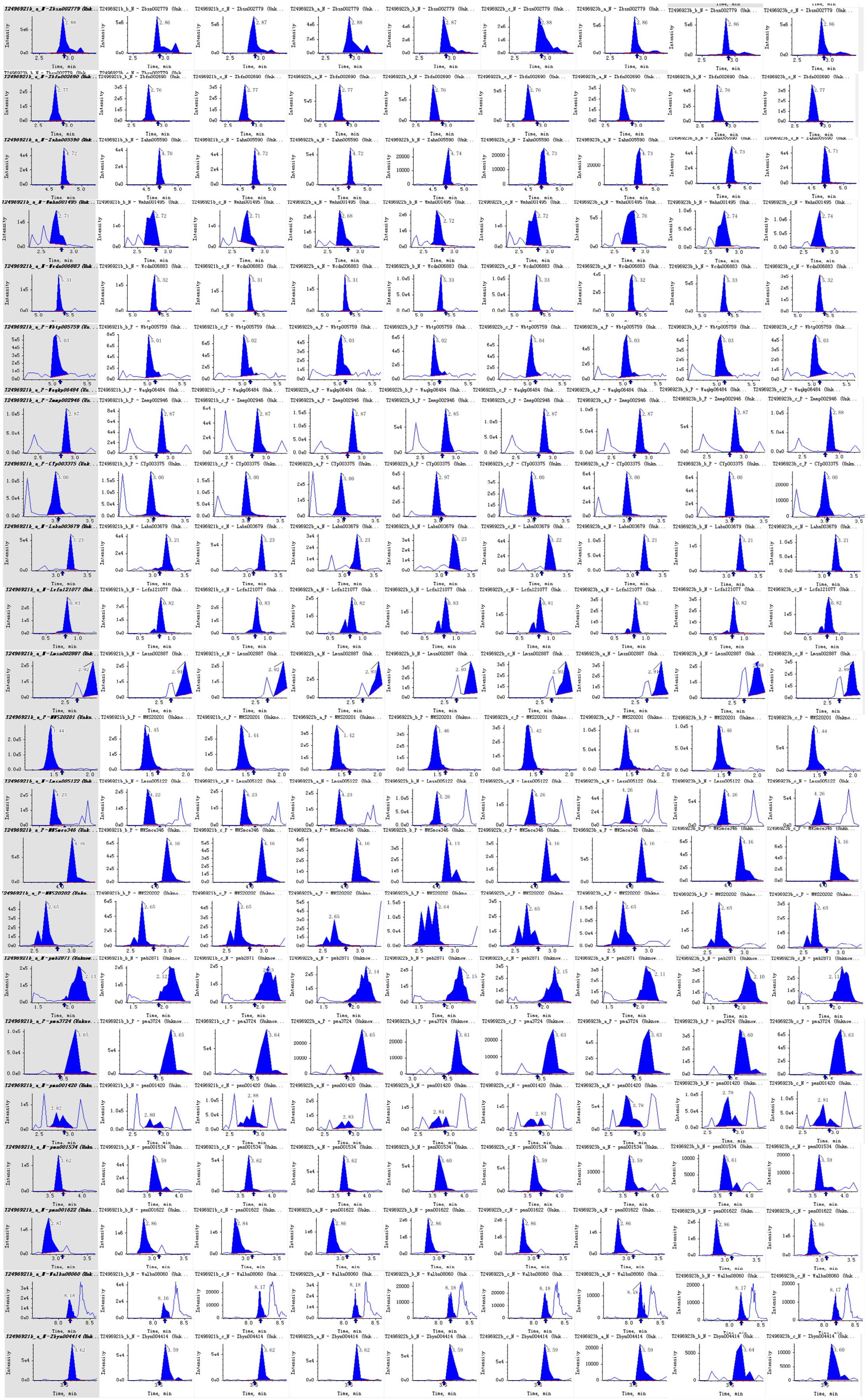

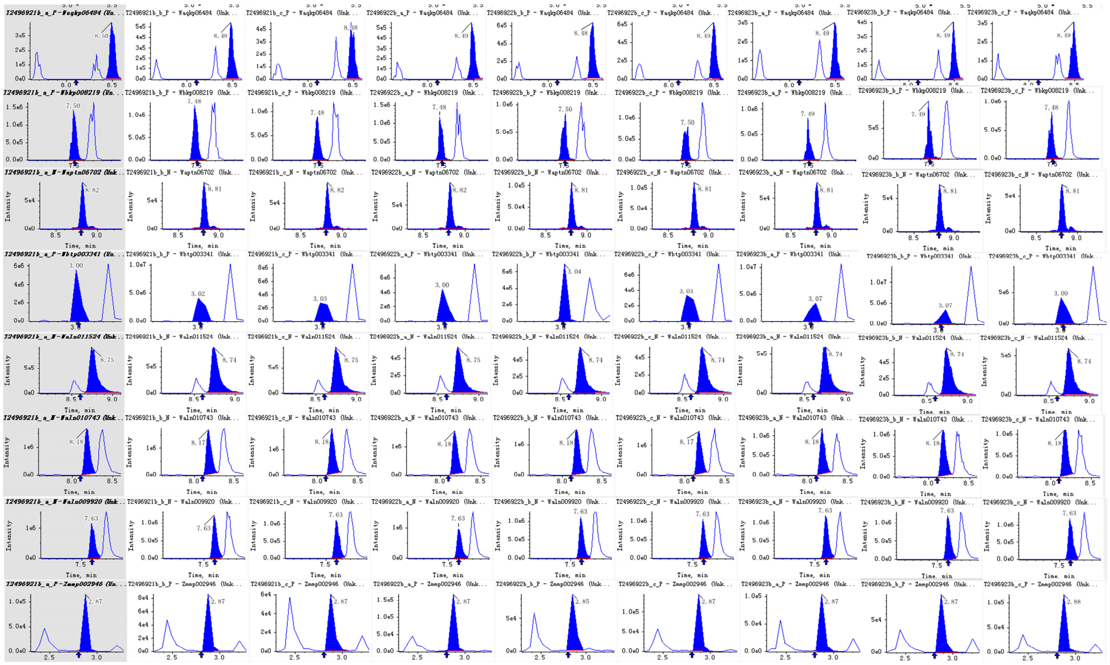

Figure S2: The UPLC-MS/MS chromatogram of 30 representative non-volatile compounds (level 1).

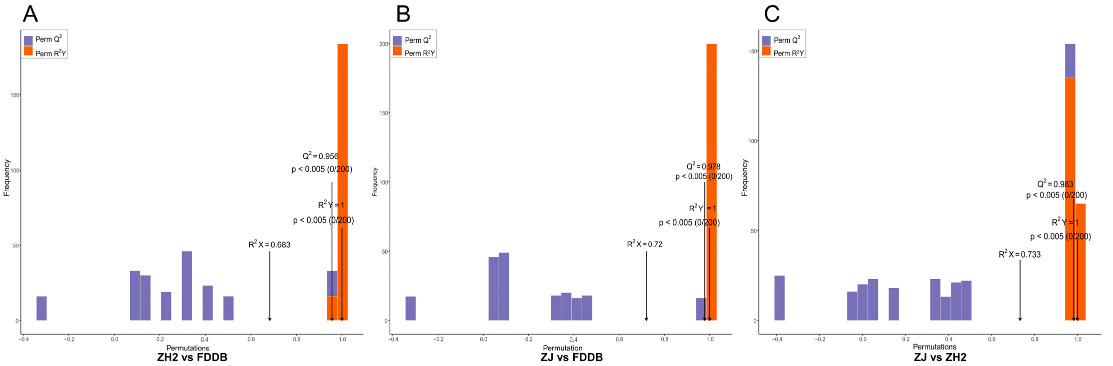

Figure S3: Permutation test of the OPLS-DA model of the non-volatile compounds; (**A**) ZH2 vs FDDB; (**B**) ZJ vs FDDB; (**C**) ZJ vs ZH2.

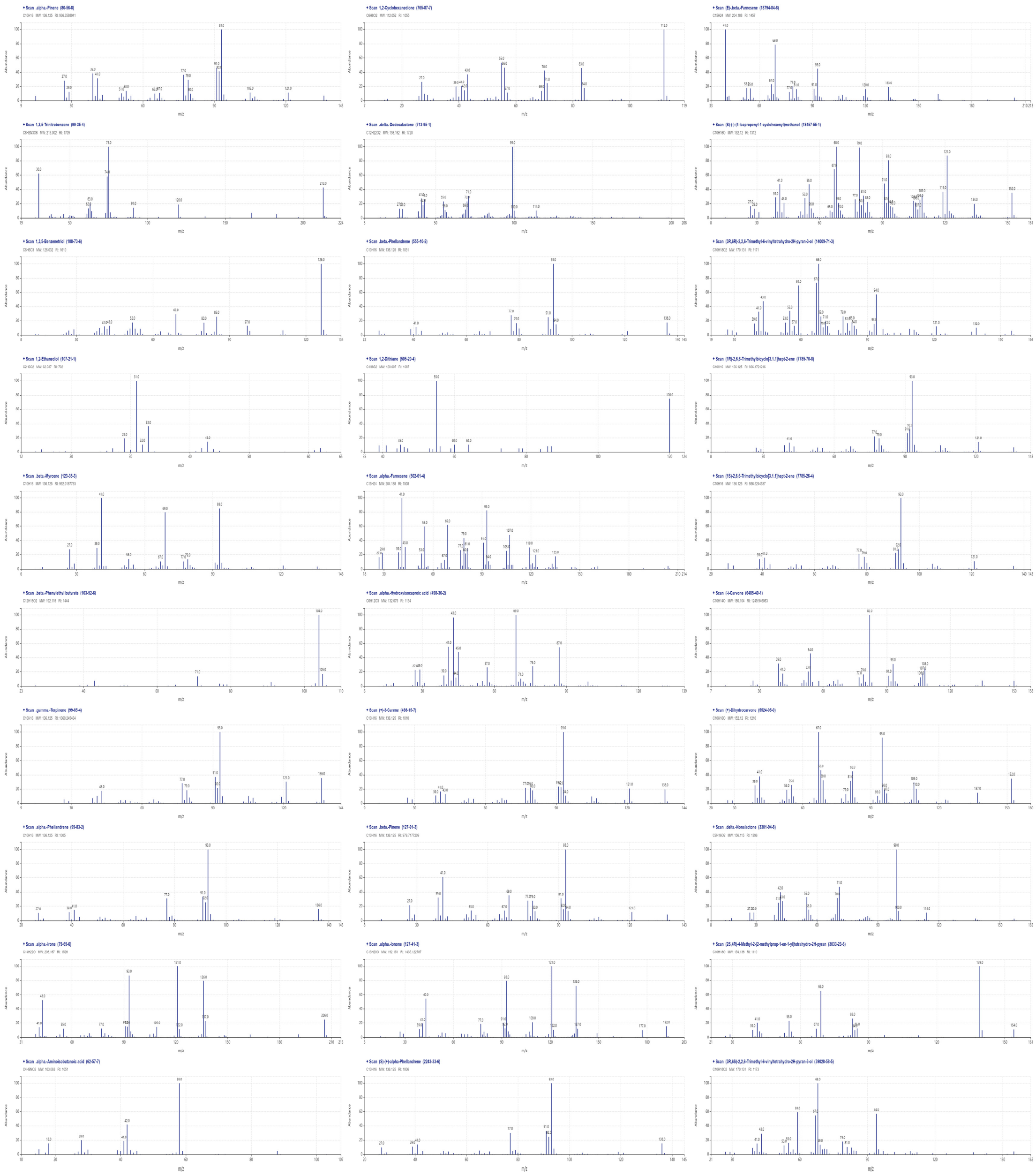

Figure S4: The MS/MS spectra of 30 representative volatile compounds.

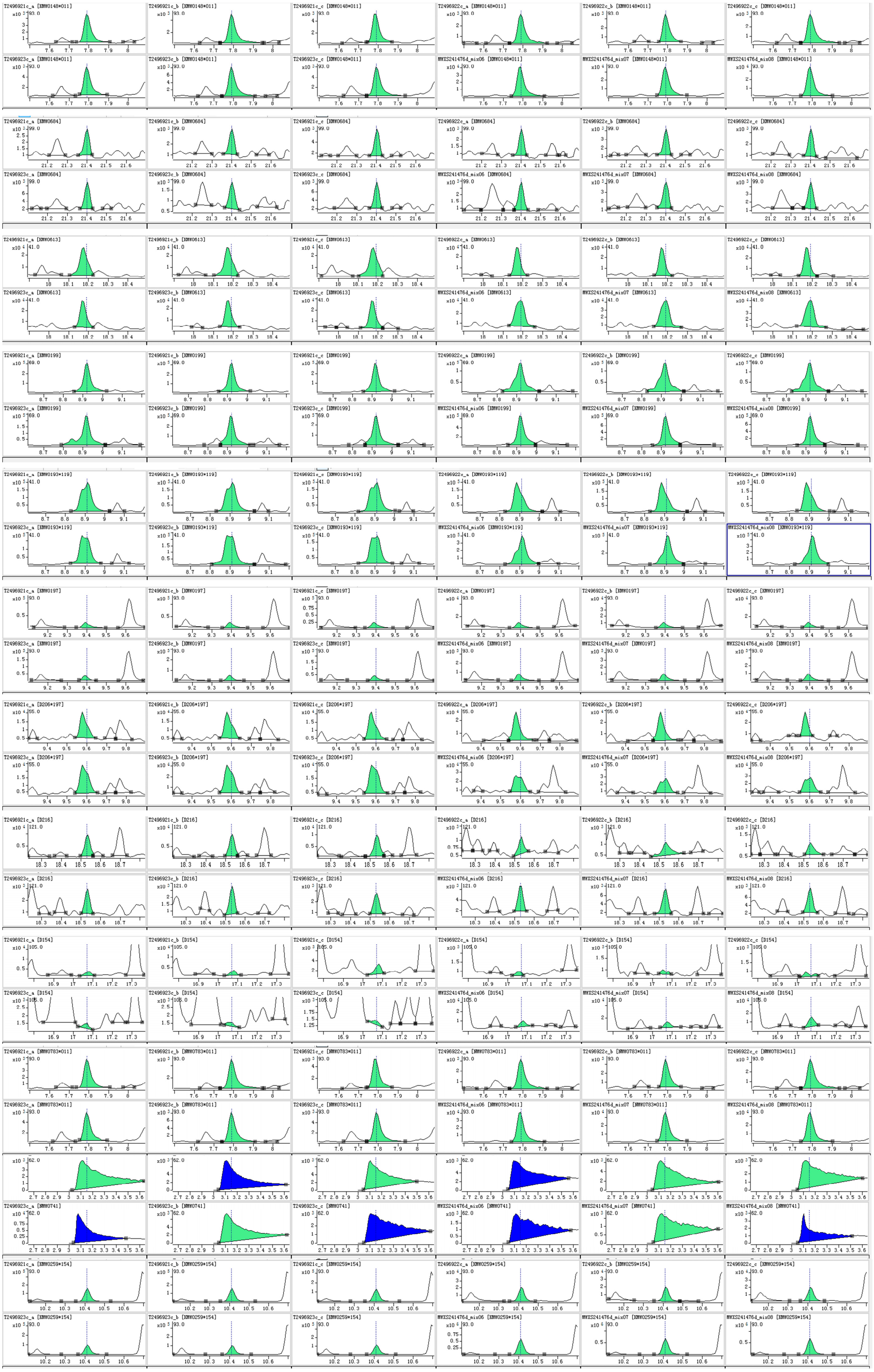

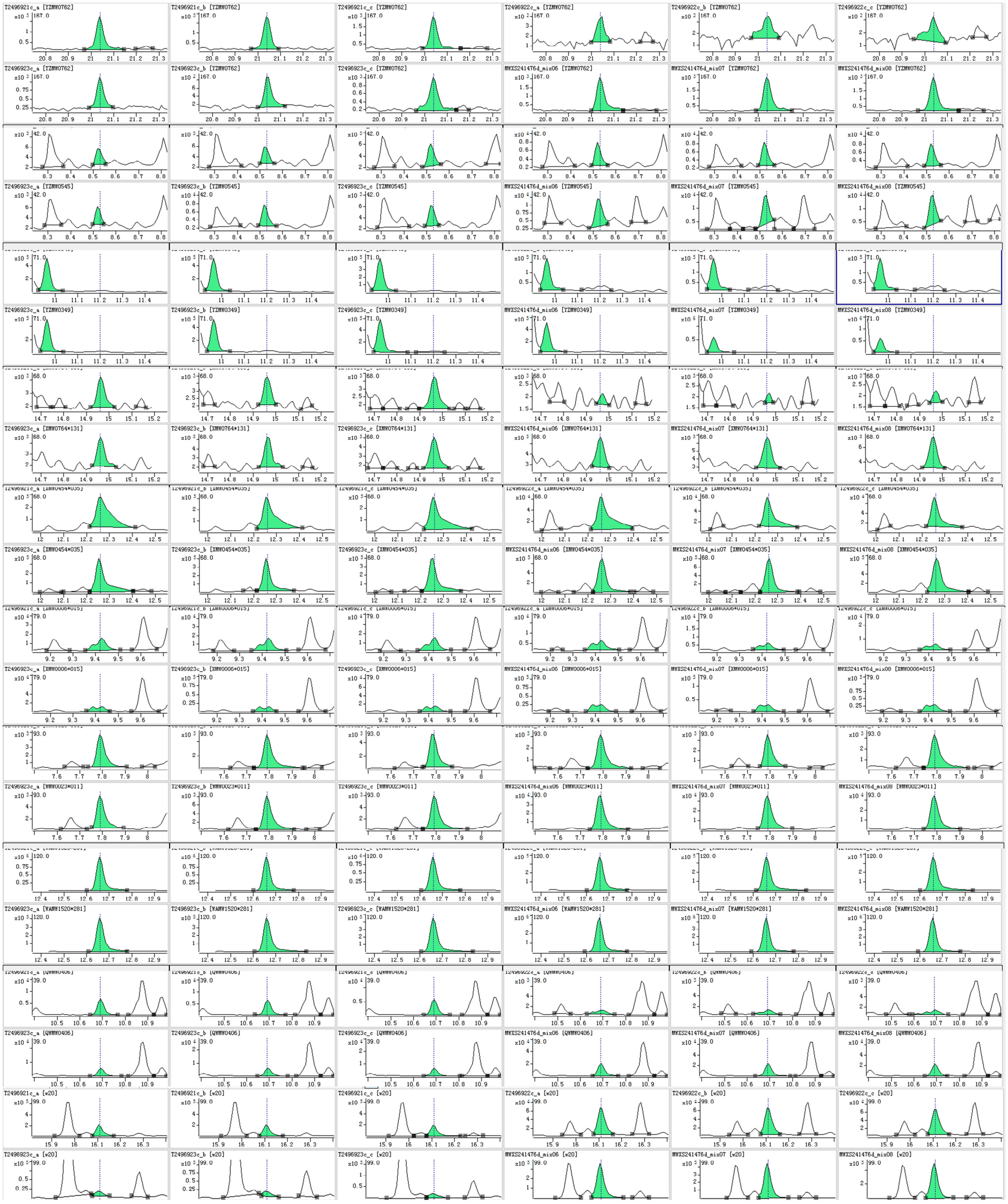

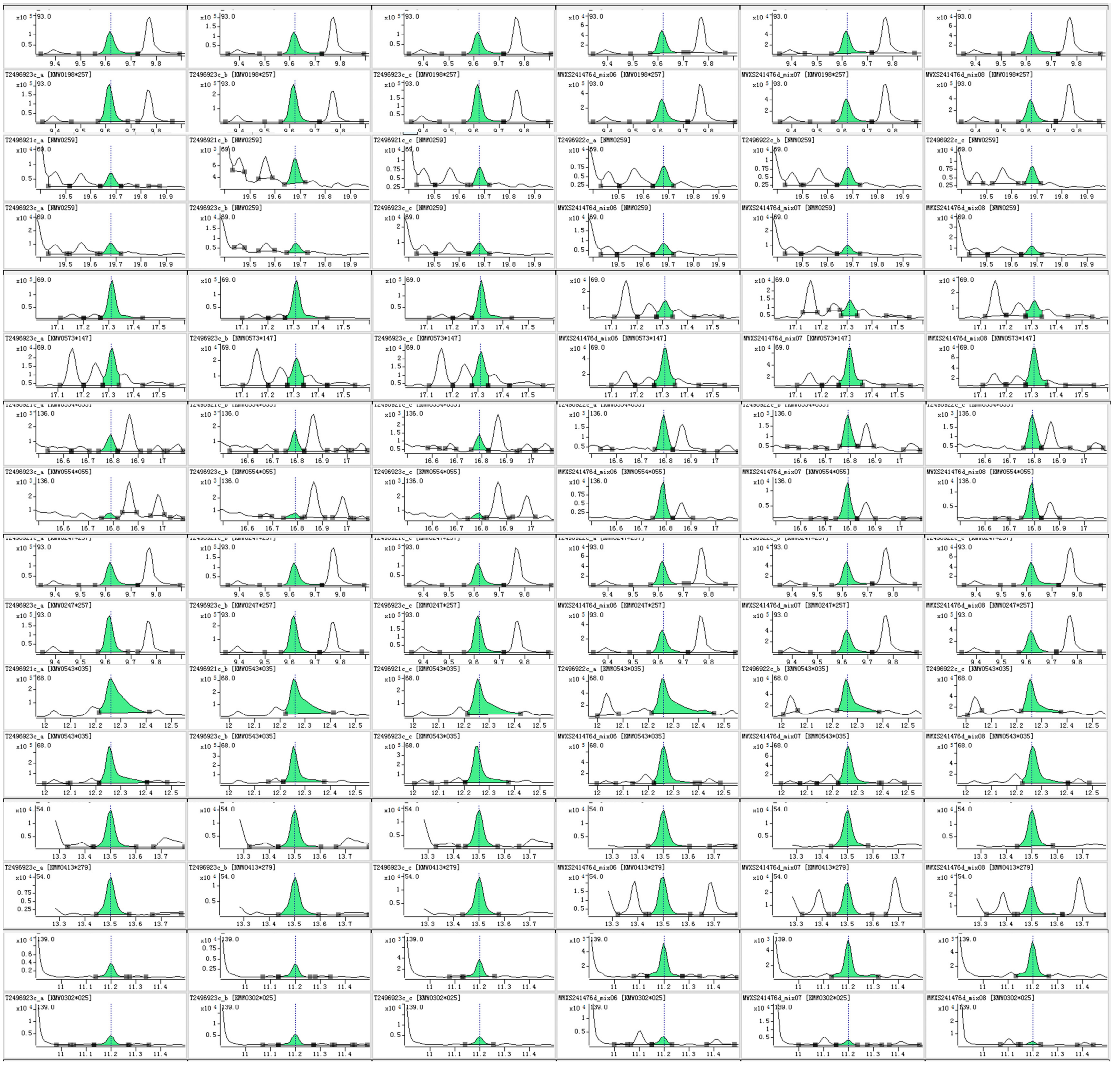

Figure S5: The GC-MS/MS chromatogram of 30 representative volatile compounds.

Table S1: The MRM ion pairs (Q1 and Q3) for non-volatile compounds

Index	Q1 (Da)	Q3 (Da)	Compounds	Class I
Zmmp002946	295.13	120.08	(3-(carboxyamino)-2-methylpropanoyl)phenylalanine	Amino acids and derivatives
Zmln000668	493.12	169.01	Monogalloyl-diglucose	Phenolic acids
Zmdp000292	189.14	70.07	Arginine methyl ester*	Amino acids and derivatives
Zaln004057	335.08	179.03	4-caffeoylshikimic acid*	Phenolic acids
Wmmp000175	635.09	277.03	pterocaryaninB	Tannins
Wcdn006883	357.13	151.04	(+)-epipinoresinol*	Lignans and Coumarins
Wbtp005759	303.05	303.05	1,2,5,7,8-pentahydroxy-3-methylanthracene-9,10-dione*	Quinones
Wbmp002283	146.06	118.06	A-hydroxyquinoline	Alkaloids

Waln003168	341.09	195.05	5-O-p-Coumaroyl Galactonic Acid	Phenolic acids
Wafn011571	295.23	195.14	alpha-Hydroxylinoleic acid*	Lipids
Wafn002081	285.06	153.02	Dihydroxybenzoyl xyloside	Phenolic acids
Sazp003601	387.17	207.11	Tuberonic acid glucoside*	Organic acids
pmn001367	315.07	153.02	Protocatechuic acid-4-O-glucoside*	Phenolic acids
pme3337	384.12	252.07	Succinyladenosine	Nucleotides and derivatives
pme3188	323.03	211.00	Uridine 5'-monophosphate	Nucleotides and derivatives
pme1611	433.11	313.07	Isohemiphloin	Flavonoids
pmb0681	403.10	367.08	Apigenin-8-C-Arabinoside*	Flavonoids
MWStz746	535.14	481.11	Apigenin 6-C-alpha-L-arabinopyranosyl-8-C-beta-D-xylopyranoside*	Flavonoids
MWStz475	535.15	499.13	Apigenin 6,8-di-C-alpha-L-arabinopyranoside*	Flavonoids
MWStz430	595.16	271.06	Apigenin 7,4'-diglucoside	Flavonoids
MWSslk254	595.17	287.06	Kaempferol-3-O-glucorhamnoside*	Flavonoids
MWSHY0181	433.11	313.07	Apigenin-8-C-Glucoside (Vitexin)*	Flavonoids
MWSHY0177	443.10	139.04	Catechin gallate	Flavonoids
mws2186	435.09	303.05	Avicularin(Quercetin-3-O-α-L-arabinofuranoside)*	Flavonoids
mws1422	275.09	107.05	Epiafzelechin	Flavonoids
Lmmp002143	567.10	319.05	Gossypetin-3-O-(6''-malonyl)glucoside*	Flavonoids
Lmmp001947	481.10	319.05	Gossypetin-3-O-glucoside*	Flavonoids
Lcsp013328	324.29	62.06	linoleoyl ethanolamine	Lipids
Lcfp121212	286.13	106.07	o-aminobenzylbeta-D-glucopyranoside	Alkaloids
CYn002543	425.04	241.00	Methylgallic Acid 3-(6''-Sulfate)Glucoside	Phenolic acids

Table S2: The SIM ion pairs (Quantitative and Qualitative) for volatile compounds

Index	Quantitative ion	Qualitative ion	Compounds	Class I
KMW0234*016	119.00	134.00	p-Cymene	Terpenoids
KMW0297*088	59.00	94.00	trans-Linalool oxide (furanoid)	Heterocyclic compound
KMW0299*025	139.00	69.00	trans-Rose oxide	Terpenoids
KMW0366*313	67.00	82.00	Butanoic acid, 3-hexenyl ester, (Z)-	Ester
KMW0389*092	95.00	110.00	endo-Borneol	Terpenoids
KMW0418	135.00	107.00	(1R)-(-)-Myrtenal	Aldehyde
NMW0037*023	103.00	70.00	Butanoic acid, 3-methyl-, 3-methylbutyl ester	Ester
NMW0198*289	156.00	141.00	Naphthalene, 1,2-dimethyl-	Ether
NMW0259	69.00	126.00	1,3,5-Benzenetriol	Phenol
NMW0794*250	91.00	134.00	Benzene, (1-methylpropyl)-	Aromatics
QWMW0216	41.00	72.00	3-Decanone	Ketone
QWMW0607	85.00	113.00	3-Nonanone	Ketone
WAMW0327*295	85.00	103.00	Butanoic acid, 3-methyl-, butyl ester	Ester
WAMW1674	74.00	43.00	Urea, methyl-	Nitrogen compounds
WAMW2190*095	71.00	81.00	dl-Menthol	Terpenoids
WMW0046	57.00	41.00	trans-2-Undecen-1-ol	Alcohol
WMW0071	182.00	75.00	Trisulfide, dipropyl	Sulfur compounds
WMW0081*013	107.00	136.00	cis-2-(2-Pentenyl)furan	Heterocyclic compound
XMW0127*016	119.00	134.00	o-Cymene	Terpenoids
XMW0186*139	95.00	43.00	3,5-Octadien-2-one, (E,E)-	Ketone
XMW0416	79.00	136.00	1,3-Cyclohexadiene, 5-butyl-	Hydrocarbons
XMW0958	155.00	43.00	1-Iodoundecane	Halogenated hydrocarbons

XMW0965*143	119.00	93.00	(1S,5S)-2-Methyl-5-((R)-6-methylhept-5-en-2-yl)bicyclo[3.1.0]hex-2-ene	Hydrocarbons
XMW1142*040	107.00	135.00	.alpha.-Thujenal	Aldehyde
XMW2237	77.00	106.00	p-Aminotoluene	Amine
XMW3422	67.00	94.00	n-Pentylpyrazine	Heterocyclic compound
YZMW0115	108.00	107.00	Butanoic acid, 2-methylphenyl ester	Ester
YZMW0349	71.00	43.00	.alpha.-Hydroxyisocaproic acid	Acid
YZMW0545	42.00	41.00	.alpha.-Aminoisobutanoic acid	Acid
YZMW0707*271	115.00	116.00	1-Naphthalenol	Phenol
